# Advances in Radiobiology of Stereotactic Ablative Radiotherapy

**DOI:** 10.3389/fonc.2020.01165

**Published:** 2020-08-07

**Authors:** Bin Qiu, Abudureyimujiang Aili, Lixiang Xue, Ping Jiang, Junjie Wang

**Affiliations:** Department of Radiation Oncology, Peking University Third Hospital, Beijing, China

**Keywords:** radiotherapy, radiobiology, stereotactic ablative radiotherapy, radiosensitivity, oncology

## Abstract

Radiotherapy (RT) has been developed with remarkable technological advances in recent years. The accuracy of RT is dramatically improved and accordingly high dose radiation of the tumors could be precisely projected. Stereotactic radiosurgery (SRS) and stereotactic body radiotherapy (SBRT), also known as stereotactic ablative radiotherapy (SABR), are rapidly becoming the accepted practice in treating solid small sized tumors. Compared with the conventional fractionation external beam radiotherapy (EBRT), SABR with very high dose per fraction and hypo-fractionated irradiation yields convincing and satisfied therapeutic effects with low toxicity, since tumor cells could be directly ablated like radiofrequency ablation (RFA). The impressive clinical efficacy of SABR is greater than expected by the linear quadratic model and the conventional radiobiological principles, i.e., 4 Rs of radiobiology (reoxygenation, repair, redistribution, and repopulation), which may no longer be suitable for the explanation of SABR's ablation effects. Based on 4 Rs of radiobiology, 5 Rs of radiobiology emphasizes the intrinsic radiosensitivity of tumor cells, which may correlate with the responsiveness of SABR. Meanwhile, SABR induced the radiobiological alteration including vascular endothelial injury and the immune activation, which has been indicated by literature reported to play a crucial role in tumor control. However, a comprehensive review involving these advances in SABR is lacking. In this review, advances in radiobiology of SABR including the role of the 4 Rs of radiobiology and potential radiobiological factors for SABR will be comprehensively reviewed and discussed.

## Introduction

Radiotherapy (RT) is a fundamental therapeutic approach for all kinds of tumors which is carried out in ~60–70% of newly diagnosed cancer patients or adjuvant/new adjuvant for surgery and palliative modality (1, 2). The advent of new radiation delivery technologies, for example, intensity modulated radiation therapy (IMRT), volumetric intensity modulated arc therapy (VMAT), and image guided radiation therapy (IGRT), has led to an evolving capability to maximize dose conformity. Accordingly, high-dose radiation can be precisely projected to tumors (3). The fractionation paradigm of RT shifts from the conventional multifractionated radiation to hypo-fractionated radiation (3, 4). Hence, stereotactic radiosurgery (SRS) and stereotactic body radiotherapy (SBRT), also known as stereotactic ablative radiotherapy (SABR), have been rapidly becoming the established mainstream of clinical practice, especially for small sizes or early stage cancer (5–7). Impressively, the clinical efficiency of SABR is greater than expected by the linear quadratic model and the conventional radiobiological principles of 4 Rs, including “repair of sublethal cellular damage,” “redistribution of cells within the cell cycle,” “reoxygenation of the surviving cells,” and “repopulation of cells after radiation,” which may no longer be suitable to account for the killing effects of SABR (8–10). Most likely, the underlying mechanisms of tumor response to radiation might also be involved in the intrinsic radiosensitivity and new radiobiological factors, e.g., vascular damage (10, 11). Here, the roles of 4 Rs of radiobiology and potential new radiobiological factors for SABR will be reviewed.

## Conventional Fractionation RT and the 4 Rs of Radiobiology

After the discovery of X-rays in 1895, Roentgen et al. first irradiated cancers with whole radiation dose delivered in single fraction (12). However, by the 1930s, it was demonstrated that RT with multifractionated radiation was more effective than single fraction radiation, which created a beneficial differential effect between cancer and normal cells (13). In 1934, Coutard et al. proposed a fractionation scheme of 200 Rontgen per fraction and 5 fractions per week, which was converted into the standard contemporary 2 Gy per fraction a day and 5 days a week scheme (3, 14). These early clinical and radiobiological observations led to the development of basic principle and fractionated practice of conventional EBRT with 25–35 fractions in 4–6 weeks. This external beam radiotherapy (EBRT) pattern achieved great success in treating epithelial tumors (e.g., laryngeal, nasopharyngeal, and skin cancer), which laid the foundation of conventional multifractionated radiotherapy (3).

However, in conventional radiation dose range (generally 1–5 Gy per fraction with total doses of 60–70 Gy), the radiosensitivity of tumor varies greatly from tumor to tumor of different tissues. Moreover, the RT for all tumors with constant dose, fractions, and treatment cycle obviously lacked personalization and pertinence due to the differences in pathological types, differentiation, and biological behavior of tumors. Consequently, the expected effect is hardly achieved under the conventional multifractionated EBRT for the treatment of radio-resistant tumors, e.g., lung adenocarcinoma, pancreatic cancer, liver cancer, melanoma, renal cancer, and soft tissue sarcoma. Therefore, the substantial evidence gathered over several decades indicated that this may not be the optimal approach for all targetable tumors (6).

Clinical and radiobiological research revealed that the responses of tumor and normal tissues to conventional multifractionated radiotherapy are commonly governed by several radiobiological principles at both cellular and histological levels. Owing to the low-dose fraction and long-term conventional EBRT, tumor cells could not be completely killed, and therapeutic resistance to radiation usually occurs. The 4 Rs of radiobiology aforementioned were initially described by Withers on the radiobiological response to conventional EBRT, which constituted the cornerstone of radiobiological theory of conventional EBRT (8, 10). In the setting of conventional EBRT, “reoxygenation” and “redistribution” increase the radiosensitivity of tumor cells and thus contribute to the killing effect of RT. “Repair” and “repopulation” of tumor cells are associated with the occurrence of radiation resistance which decrease the radiosensitivity of tumor cells and hence greatly decrease the killing effects of RT (10).

## SABR

### The Discovery of SABR

In 1951, Leksell (15) first utilized gamma rays to focus radiation on intracranial targets and described the concept of SRS. Then Gamma-knife, as the first radio-surgical device, was developed and introduced in 1967 at the Karolinska Institute (16). Although SRS was introduced originally for functional neurosurgery, it was soon applied in the RT for intracranial metastatic tumors, which yielded the promising efficacy comparable to surgery (17, 18). Compared with the conventional EBRT, SRS with single fraction/hypofractionation and total doses of 15–25 Gy achieved high local control of tumor (17–19). The implementation of SBRT was delayed as a result of facing challenges in both physiological motion and confidence in tumor-targeted therapy (20). In the 1990s, CyberKnife (Accuray, Sunnyvale, USA), the highly complex radiosurgery system, was invented at Stanford University (21). Along with the advances in irradiation technology, the paradigm and efficacy of RT have undergone the radical changes. Until 2002, Timmerman et al. first used the stereotactic ablation radiosurgery for the treatment of inoperable early stage lung cancer, which was initially called “extracranial stereotactic radio-ablation” (22, 23). Afterwards, SBRT was defined by the American Society for Therapeutic Radiology and Oncology (ASTRO) (22, 23). The concept of SABR was first proposed in 2011 by Loo et al. (24), which included SRS and SBRT for the treatment of solid tumors. SABR with high dose per fraction and hypo-fractionated radiation yields the convincing and satisfied therapeutic effects with low toxicity, since tumor cells are directly ablated in response to high-dose radiation (7, 25). Therefore, SABR may overcome the dilemma of insensitivity to tumors which are resistant to conventional EBRT.

### Characters of SABR

SABR is delivering such large doses per fraction to tumors mainly owing to the remarkable advances in tumor imaging, dosimetry, and radiation delivery technology. Compared with conventional fractionation RT, SABR yields several characteristics, described as the following.

#### Focalized Conformal Irradiation

Owing to the advances of treatment planning system and multi-leaf collimators driven by computerized algorithms, focalized 3D conformal RT (3D-CRT) for target regions and organs at risk could be achieved, namely dose painting or dose sculpting RT (26). Gamma-knife achieved focalized and conformal irradiation using collimator whereas CyberKnife used manipulator tracking and dynamical irradiating (27, 28). At present, RT has entered the 3-dimensional/4-dimensional era with ablative efficacy that rivals surgery, different from previous 2-dimensional conventional EBRT (3, 4). Compared with conventional multifractionated EBRT, the optimal iso-dose of SABR sculpting in the tumor volume is dramatically increased with less sparing margins surrounding normal tissues (7).

#### Image Guidance

Image guidance has also evolved from ultrasound, interactive X-ray, cone-beam CT (CB-CT), and CT to MRI guidance (4, 29). Ultrasound guidance is mainly used for RT of prostate cancer. Interactive X-ray is used, along with the stereospecific system, in CyberKnife for tracking fiducials previously implanted into the tumor. CT-based simulation and planning allow better radiation dose distributions, which are the major image guidance in radiotherapy (IGRT) of linear accelerator (3). MRI-guided clinical application with MRIdian (ViewRay Inc., Oakwood Village, OH) was reported in 2012 and MRI-Linac system was developed in 2016 (29–31). The major advance of image guidance solves the shifting caused by the movement of target organ during irradiation, greatly improves the irradiation accuracy, and reduces the damages to surrounding normal tissues.

#### High Dose Per Fraction and Short Course of Treatment

High dose per fraction and short course of treatment can be achieved when the accuracy of irradiation is granted, while geometrically sparing the innocent normal tissues (20). High-dose radiation to small volume targets in a single or small number of fractionations could be precisely projected by stereotactic focus under image guidance (32, 33). SABR was delivered with high dose per fraction in a relatively short course (SBRT delivers 40–60 Gy in 1–5 fractions and SRS irradiates lesions with 18–25 Gy typically in a single fraction) (10, 11). Encouraging efficacy of SABR was achieved in the treatment of early-stage lung cancer, liver cancer, and other previously considered “radioresistant” tumors, such as metastasis of lung, liver, and spinal cord (5, 34–36).

#### Application in Parallel Organs

Owing to the ablative property, SABR was mainly applied in parallel organs, such as lung, liver, pancreas, kidney, and prostate (37, 38) which are dose-volume dependent and can tolerate the relatively high-dose radiation (39). Serial organs, such as esophagus, stomach, and rectum, are considered to be not suitable for large fractional radiation, since all downstream function may be disrupted when a section of serial organs is damaged anywhere along its length (40, 41).

### Proposed Classification for SABR

Since the extended boundary for the formation of planning target volume (PTV) is inconsistent in varied SABR technology (e.g., Gamma-knife, Liner-accelerator, CyberKnife, and TomoTherapy system), the clinical efficacy and its side effects are quite variant. Therefore, we proposed a classification for SABR based on each equipment and technology mentioned above and classified SABR into Gamma-knife-SABR (G-SABR), Liner-accelerator-SABR (L-SABR), CyberKnife-SABR (C-SABR), Tomo-SABR (T-SABR), and Proton-SABR (P-SABR), which was thought to be convenient for communication and comparison of the clinical outcomes among these technologies. Meanwhile, taking advantage of the inverse-square law, Brachytherapy (BT) driven by high-precision imaging and planning offers an intrinsically conformal dose distribution, which facilitates dose escalation (3). Stereotactic ablative brachytherapy (SABT) has been achieved nowadays, including high-dose rate SABT (H-SABT) and low-dose rate SABT (L-SABT), and may also be classified into SABR technology.

### The 4 Rs of Radiobiology in SABR

#### SABR and Repair

Tumor cells with lethal damage will lead to DNA breakage and cell death under conventional EBRT. However, tumor cells with sublethal damage/potential lethal damage will repair and continue to proliferate after a certain period of adjustment owing to the inadequate doses radiation, which results in tumor recurrence and metastasis (42, 43). Repair compromises the efficiency of radiation and reduces the radiosensitivity of tumors, as radiosensitivity correlates with the number of residual unrepaired DNA double strand breaks (44, 45). In the setting of SABR, high-dose radiation per fraction is applied and total doses are delivered in 2–5 times fractions within a relatively short period, inducing more necroptosis than apoptosis (46, 47). Therefore, the repair of tumor cells is almost impossible or at a very low incidence. Accordingly, the majority of tumor cells will suffer from lethal damage leading to cell death (10, 47).

#### SABR and Redistribution

After irradiation, tumor cells at G0 stage of cell cycle will accelerate into G2/M stage for replenishment or rebalancing (radiation-induced G2/M arrest) (48, 49). Tumor cells at G2/M stage are highly sensitive to radiation. During conventional EBRT, the sensitivity of radiation is potentially enhanced, as the proportion of tumor cells at G2/M stage increases (50). Therefore, redistribution of cell cycle improves the killing ability of conventional multi-fractionated EBRT (50). While in the setting of SABR, the cell cycle is completely blocked at all stages after single higher-dose ablation radiation (e.g., >20 Gy). Therefore, it is impossible for tumor cells' redistribution since both sensitive and insensitive tumor cells are directly killed (48).

#### SABR and Reoxygenation

Given that oxygenated tumor cells are sensitive to radiation during conventional EBRT, tumor cells in hypoxic state will reoxygenate and be killed by radiation. Thus, reoxygenation enhanced the killing effects in the setting of conventional fractionated EBRT (10). Reoxygenation may be reduced owing to the relative short duration of SABR. Furthermore, tumor hypoxia may persist after vascular injury caused by SABR (11, 51). In such cases, additional radiation dose boost may offer the solution to overcome the state of hypoxic radioresistance (52). Both oxygenated and hypoxic cells are ablated by high-dose radiation under SABR, resulting in highly efficient tumor killing.

#### SABR and Repopulation

The sensitive tumor cells quickly enter the apoptosis state under conventional EBRT, leading to cell populations' unbalance. In the beginning of homeostasis, tumor cells at stationary stage will proliferate to compensate for the loss of cell populations. Repopulation of tumor cells usually occurs in 2–3 weeks after conventional fractionated EBRT, depending on the fractionated radiation doses, total doses, and pathological types with increase of radiation resistance and decrease of killing effects (10). SABR treatment scheme is usually within 2–5 fractions and completed within 1 week with no time to spare the tumor cells to start the repopulation process (53, 54).

Therefore, the 4 Rs of radiobiology contributes little to the killing effects of SABR, as the majority of the tumor cells are ablated. The different patterns of intrinsic radiosensitivity among cells and tissues may play an important role in tumor response, which was demonstrated by Bergonie and Tribondeau in 1906 (3). Intrinsic radiosensitivity of tumor cells represents a component attributing to the therapeutic outcome of conventional multifractionated EBRT whereas further investigation is needed in SABR (45). Based on the 4 Rs of radiobiology, 5 Rs of radiobiology was first proposed by Steel et al. (55), emphasizing the intrinsic radiosensitivity of tumor cells, which is correlated to the responsiveness of tumors to radiation. Martin Brown et al. favored the 5 Rs of radiobiology; however, he raised the question whether there are any new radiobiological factors that have not been defined yet (9, 10).

### Dose-Effect Relationship Models in SABR

Linear Quadratic Model (LQ Model) is applicable to the calculation of iso-effect doses in treating cancers with conventional EBRT (56). The ratio of alpha to beta (α/β) reflects the extent of biological effects on tissues and cells affected by fractionated radiation doses (57). α/β in early-responsive tissue/tumor is larger (about 10 Gy) than that of late-responsive tissue/tumor (about 3 Gy) (58). The prerequisite of LQ model application is complete oxygenation of tumor cells during radiation with the fractional dose of lower than 1–6 Gy (10). When fractional dose is higher than 8–10 Gy, the LQ model is inappropriate to predict the effects induced by radiation (56, 59). Overprediction of the potency and toxicity of SABR by LQ model made clinicians hesitate to adopt the efficacious and well-tolerated therapeutic option (60, 61). However, some clinical studies find that LQ model actually underestimates tumor control by SABR (59, 61). In 2004, in order to precisely describe the biological effects of high dose per fraction, Guerrero and Li suggested to refine LQ model and the modified LQ model (MLQ) was proposed (62). In 2008, Park et al. (60) introduced the Universal Survival Curve model (USC model), which integrated LQ model with multi-target model, and incorporated the effects by both low dose and high dose radiation. The concept of inflection point dose is proposed in USC model, in which RT under inflection point dose is adapted to LQ model and radiation above inflection point dose is adapted to USC model (60). In 2010, Wang et al. (63) introduced the general LQ model (gLQ model), which involves all dose range. However, the relationship between biological effects of high dose radiation per fraction and the actual clinical efficacy could not be comprehensively explained by these models since indirect effects such as radiation-induced injury of blood vessels are not included (63).

### Potential Radiobiological Factors of SABR

As a highly targeted technique, SABR delivers high-dose radiation to ablate tumors directly (6). The latest clinical studies have confirmed that SABR not only ablates tumor cells directly but also induces indirect effect, including vascular endothelial injury and immune activation. Indirect tumor cell death by SABR may play a crucial role in the tumor killing (64).

#### Vascular Endothelial Injury

As a homeostatic factor, endothelial apoptosis regulates angiogenesis-dependent tumor growth, which only occurs at radiation doses above ~8–11 Gy (65). Other studies also found obvious vascular injury under high-dose radiation, especially above 10 Gy, which induced hypoxia, acidification of tumor microenvironment, and indirect death of tumor cells (66, 67). High-dose radiation delivered by SABR increased vascular permeability and apoptosis through the ceramide pathway (68). Vascular endothelial injury exacerbated platelet aggregation and thrombosis formation, which further blocked the blood vessel. High dose radiation induced blood vessel injury and ischemia, further leading to tumor necrosis. Consequently, anti-tumor effect of radiotherapy was enhanced (65).

#### Immune Activation

RT stimulates responses not only at the treatment site but also at the non-irradiated and remote tumor deposits, which is called “abscopal effect” (69). RT directly or indirectly activates inflammatory cytokine, e.g., IL-1 and TNF, recruits immune cells, resulting in an intense CD8(+) T-cell tumor infiltrate and a loss of myeloid-derived suppressor cells (70), tumor cells are ablated and tumor antigens are substantially released under high dose radiation, leading to immunogenic cell death and further waterfall-like release of tumor necrosis antigens and adenosine triphosphatase (ATP). The activation and release of these substances enhance the human immune responses and immune cells recruitment to the microenvironment (71). Based on the elucidated immune mechanisms, the combination of radiotherapy with immune therapy has been developed for anti-tumor therapeutic approach (69).

## Discussion

Given the rapid innovative technological advances, RT entered a new era of ablative radiotherapy with high-dose radiation per fraction and short course, the role of 4 Rs of radiobiology is facing challenges in the setting of SABR. In addition, along with the directly ablative effect induced by SABR, indirect effects induced by vascular endothelial injury and immune activation should be noted. Moreover, the concept of radiation effect on metabolic microenvironment is emerging (72, 73). Therefore, in the era of ablative radiation, the study of radiobiology should cover tumor cells, immune cells, and metabolic microenvironment (72, 73). In the future, additional factors, e.g., the number and proportion of differentiated immune cell, differentiation stages, and tumor microenvironment, should be considered in the prescription of dose and fraction of RT. New technology, e.g., single-cell sequencing, metabolic imaging, and artificial intelligence (AI), will certainly accelerate the evolution in the therapeutic modalities of RT (74–76).

## Author Contributions

BQ, AA, PJ, and JW contributed conception and design of the study and wrote the first draft of the manuscript. BQ, AA, LX, and PJ organized the literature search. LX and PJ wrote sections of the manuscript. All authors contributed to manuscript revision, read, and approved the submitted version.

## Conflict of Interest

The authors declare that the research was conducted in the absence of any commercial or financial relationships that could be construed as a potential conflict of interest.
